# Proteomics reveals multiple routes to the osteogenic phenotype in mesenchymal stem cells

**DOI:** 10.1186/1471-2164-8-380

**Published:** 2007-10-19

**Authors:** Kristin P Bennett, Charles Bergeron, Evrim Acar, Robert F Klees, Scott L Vandenberg, Bülent Yener, George E Plopper

**Affiliations:** 1 Department of Mathematical Sciences, Rensselaer Polytechnic Institute, 110 8^th ^Street, Troy, NY 12180, USA; 2 Department of Computer Science, Rensselaer Polytechnic Institute, 110 8^th ^Street, Troy, NY 12180, USA; 3 Department of Biology, Rensselaer Polytechnic Institute, 110 8^th ^Street, Troy, NY 12180, USA; 4 Department of Computer Science, Siena College, 515 Loudon Road, Loudonville, NY 12211, USA

## Abstract

**Background:**

Recently, we demonstrated that human mesenchymal stem cells (hMSC) stimulated with dexamethazone undergo gene focusing during osteogenic differentiation (*Stem Cells Dev *14(6): 1608–20, 2005). Here, we examine the protein expression profiles of three additional populations of hMSC stimulated to undergo osteogenic differentiation via either contact with pro-osteogenic extracellular matrix (ECM) proteins (collagen I, vitronectin, or laminin-5) or osteogenic media supplements (OS media). Specifically, we annotate these four protein expression profiles, as well as profiles from naïve hMSC and differentiated human osteoblasts (hOST), with known gene ontologies and analyze them as a tensor with modes for the expressed proteins, gene ontologies, and stimulants.

**Results:**

Direct component analysis in the gene ontology space identifies three components that account for 90% of the variance between hMSC, osteoblasts, and the four stimulated hMSC populations. The directed component maps the differentiation stages of the stimulated stem cell populations along the differentiation axis created by the difference in the expression profiles of hMSC and hOST. Surprisingly, hMSC treated with ECM proteins lie closer to osteoblasts than do hMSC treated with OS media. Additionally, the second component demonstrates that proteomic profiles of collagen I- and vitronectin-stimulated hMSC are distinct from those of OS-stimulated cells. A three-mode tensor analysis reveals additional focus proteins critical for characterizing the phenotypic variations between naïve hMSC, partially differentiated hMSC, and hOST.

**Conclusion:**

The differences between the proteomic profiles of OS-stimulated hMSC and ECM-hMSC characterize different transitional phenotypes en route to becoming osteoblasts. This conclusion is arrived at via a three-mode tensor analysis validated using hMSC plated on laminin-5.

## Background

Interest in human stem cells continues to grow amongst those interested in understanding fundamental mechanisms of development and disease progression and those interested in harnessing the differentiation potential of these cells to generate living replacements for damaged or diseased tissues. In both cases, the promise is the same: stem cells offer the potential to define and manipulate fundamental principles of cell and tissue behavior, which in turn will uncover a new set of therapeutic targets for correcting errors in cell and tissue function [1]. Human mesenchymal stem cells (hMSC) are a population of multipotent adult cells found within the bone marrow and periosteum [2] and capable of differentiating into as many as seven different cell types [3].

One bottleneck in the development of hMSC-derived therapies is our incomplete understanding of the mechanisms governing hMSC differentiation. For example, osteoblast differentiation from bone marrow progenitor cells (such as hMSC) has been described as a series of up to seven overlapping stages, each defined by a change in gene expression patterns [4]. Other studies suggest that these stages are a continuum, rather than distinct events [5-7]. Further complicating matters, hMSC committed to an osteogenic phenotype via treatment with dexamethazone retain the ability to transdifferentiate into other lineages [8]. Distinct patterns defining osteogenic differentiation of these cells have yet to emerge [9], though we and others have identified significant signaling and gene expression changes during osteogenic differentiation of hMSC [3,9-14].

To gain a better understanding of hMSC osteogenic differentiation, we previously used gene ontology analysis of protein expression profiles from hMSC, human osteoblasts (hOST), and hMSC stimulated to undergo osteogenic differentiation with osteogenic stimulant (OS) media containing ascorbic acid-2-phosphate, β-glycerophosphate, and the synthetic glucocorticoid, dexamethazone [15]. Our analysis revealed that OS-induced differentiation results in a decrease in the number of mesenchymal cell markers and calcium-mediated signaling molecules with a concomitant increase in expression of specific extracellular matrix molecules and their receptors, a process we call "gene focusing." [15,16] Second, we found that the protein expression profile of OS-induced hMSC partially overlapped with the profiles of both naïve hMSC and hOST, suggesting that OS-stimulated hMSC represent an "intermediate state" during osteogenic differentiation of hMSC. These results strongly imply that changes in the extracellular matrix (ECM) in the hMSC microenvironment have a direct impact on stem cell differentiation.

It is well known that ECM proteins, along with growth factors and hormones, play key roles during bone development. For example, during endochondral bone development, collagen II expression peaks during the chondrogenesis period while collagen I deposition is maximal during the ossification phase [17]. For decades it has been known that single point mutations in collagen I yield a lethal form of osteogenesis imperfecta (e.g., [18]). Genetic knockout of collagen II results in embryonic lethality associated with severe skeletal defects [19]. In vitro, hMSC undergo osteogenic differentiation when cultured on collagen I, fibronectin, vitronectin, or laminin-5 matrices [11,13,20], and this requires ECM interaction with specific integrin receptors [11,13,21,22]. A recent study demonstrates that osteogenic commitment of hMSC is irreversible after three weeks in culture on collagen I [10] but osteogenic differentiation induced by dexamethazone gradually diminishes in the absence of collagen I over the same time course [9]. These results suggest that stimulation of hMSC with dexamethazone and collagen I (or other ECM proteins) could induce osteogenic differentiation through different mechanisms and that these differences could be detected in the protein profiles of these different populations.

To test this idea, we used tensor analysis of protein expression profiles, annotated with gene ontologies, to uncover protein expression changes during the progression of stimulated hMSC towards fully differentiated hOST that distinguish distinct intermediary states of OS-hMSC and ECM-hMSC. Our results support the conclusion that OS- and ECM-induced hMSC are distinct intermediate states during osteogenic differentiation, and demonstrate that stimulation with the ECM proteins collagen I, vitronectin, and laminin-5 results in a more osteoblast-like phenotype than does stimulation with OS media.

## Results

To identify the proteins expressed by osteogenic differentiation of hMSC arising from stimulation with OS media and two ECM proteins (collagen I and vitronectin) and compare them to the protein expression profiles of undifferentiated hMSC; hMSC stimulated by OS media; and physiologically differentiated hOST, we performed 2D LC-MS/MS on whole-cell lysates of these cell populations. 758 distinct proteins indicated by an Entrez gene ID (GeneID) were identified by 2D LC-MS/MS in all five cell populations (= 200 pmol). To validate the approach, we also examined the expression of these 758 distinct proteins in hMSC stimulated by laminim-5 (Ln-5). To evaluate the functional significance of these differences, we accessed the GO (Gene Ontology) Chart featured by DAVID to identify significant GO Biological Process and Molecular Function categories for each of the 5 original samples and unioned them to form a set of 69 GO categories (provided in Additional File 1). Thus the data form a tensor or datacube with three modes: the first being 6 experiments, the second being the 758 GeneIDs, and the third being the 69 GOs. Each entry of the tensor contains a 0 if no proteins were found, 1 if exactly one protein was found, or 2 if multiple proteins were found corresponding to a given (sample, GeneID, GO) triplet. We developed two-way and three-way approaches for analyzing the (sample, geneID, GO) tensor. We begin with discussion of the results of the two-way approach.

### Two-way analysis

The two-way approach first reduces the three-mode tensor to a two-dimensional matrix in the (sample, GO) space and then uses a variation of the widely-used Principal Component Analysis (PCA) method in this space. Typically one would see PCA used for genomics in the equivalent of the (sample, geneID) space. Traditional PCA centers the data about the mean and then finds the series of orthogonal components that best explain the variance of the data [23]. Our novel approach reduces the data to a (sample, GO) matrix, transforms the data to make undifferentiated hMSC the origin, and then directs or forces the first component to be the difference between hOST and hMSC in the GO space. The remaining components are selected to maximize the explanation of the remaining variability subject to the constraint that they be uncorrelated (orthogonal) with each other and with the directed component. We call this approach to modeling *Directed Component Analysis (DCA)*, differing from *Principal Component Analysis *in that the offset and directed vectors are chosen based on biological process intuition as opposed to the mean vector and leading principal component, respectively.

The GO proteomic profiles of each of the samples are projected onto the DCA component space spanned by the first three components to provide clear insight into the distinct states of the three types of sample. To validate the hypotheses that ECM stimulated hMSC (ECM-hMSC) evoke similar intermediate differentiation states, we reserve one of the ECM samples – hMSC stimulated with laminin-5 (ln5-hMSC) – as a test sample. The components of DCA are constructed using only five samples: hMSC, vintronectin stimulated hMSC (vn-hMSC), collagen 1 stimulated hMSC (col1-hMSC), OS-hMSC, and hOST. The directed component accounts for 26% of the variability while the second, third, and fourth components account for 41%, 22%, and 10% of the variability, respectively, in the first five samples. The fourth component is largely noise and thus is discarded. Thus each sample is transformed to a three dimensional representation.

**Figure 1:**
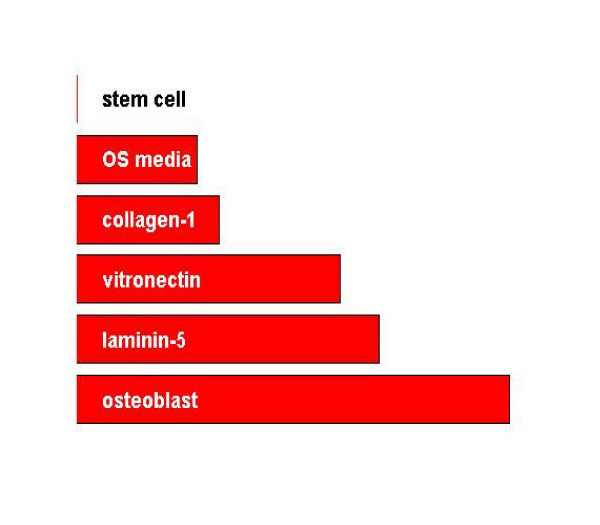
**Directed Component scores**. The directed component score ranks the samples (experiments) from stem cells to osteoblasts.

The first directed component is the direction that connects hMSC to hOST. For each experiment, *c*_*directed *_is the extent to which the experiment is similar to osteoblast along this route. These constants are plotted in Figure 1, providing an implicit ranking of experiments. We can see that stimulated hMSC fall on a spectrum from hMSC to hOST.

**Figure 2:**
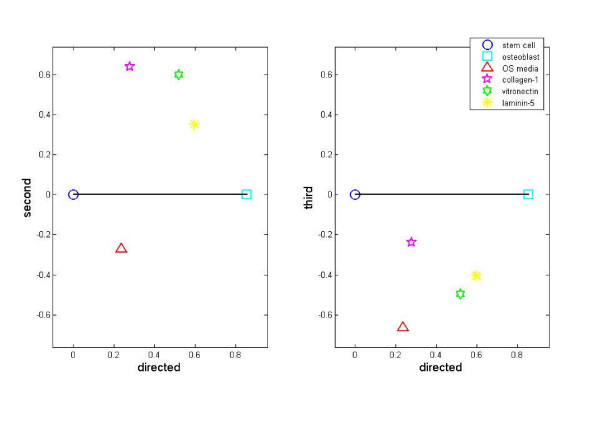
**Directed Component plots of hMSC, hOST, and stimulated stem cells**. Directed Component plots of hMSC, hOST, and stimulated stem cells demonstrate distinct lineages of ECM-hMSC vs. OS-hMSC.

We also see that the three ECM-stimulated samples cluster together including the validation sample, ln5-hMSC, which was not used to construct the components. In general the ECM-stimulated hMSC tend to more closely resemble osteoblasts than do the OS media-stimulated hMSC. Thus, the first directed component provides a characterization of how "osteoblast-like" the samples are. The two remaining components capture how the samples vary from the directed path between hMSC and hOST. The coefficients for each experiment may be plotted against each other, as in Figure 2. By construction of the directed component, the further right a population falls the closer it is to hOST. The second component plotted as the vertical axis in Figure 2 (left) shows that ECM-hMSC and hOST-hMSC form two distinct intermediate states. The ECM-hMSC cluster together at the top of the graph, meaning that they are fairly similar in many respects. OS-hMSC appears at the bottom of the plot, far from the ECM-hMSC samples. *The plot shows that the ECM-hMSC and OS-hMSC achieve distinct intermediate states along the lineage from stem cells to Osteoblasts*. Figure 2 (right) plots the third component versus the directed component. Here we see that the stimulated hMSC samples fall together and third component characterizes the variability within the intermediate states. Together the plots suggest that the stimulated hMSC represent two distinct lineages along the path from hMSC to hOST.

**Figure 3:**
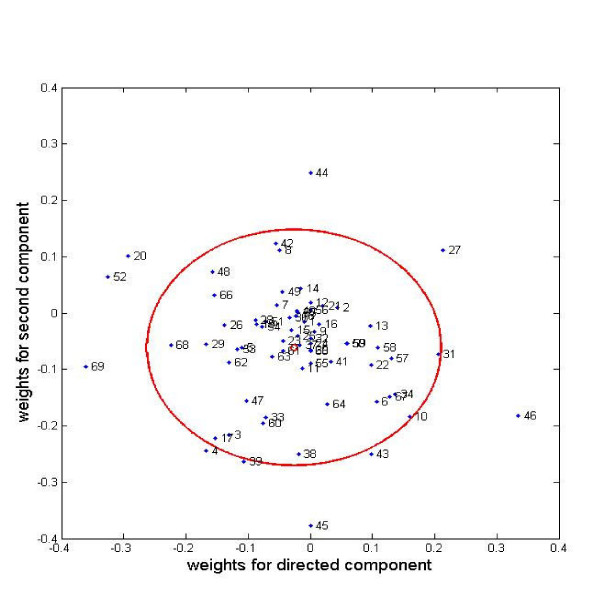
**Weights on the GOs for the directed and second components**. Weights on the GOs are plotted for the directed and second components. GOs falling outside the circle of standard deviation are critical for the components.

An interesting question is what categories have the greatest impact on the cell differentiation process. Figure 3 plots weights for these categories in the directed and second components plane. Categories near the center of the cluster (indicated by a small red circle) have little impact on the experiments, while those far from the center (and in particular outside of the circle of double standard deviation) are critical for osteogenesis.

From Figure 3, we can see that the following categories primarily characterize the route from hMSC to hOST: 20 (cytokine activity), 27 (heparin binding), 46 (oxygen and reactive oxygen species metabolism), 52 (protein kinase activity), and 69 (translation initiation factor activity). The following categories primarily distinguish between the OS- and ECM-stimulated intermediary states: 43 (oxidoreductase activity, acting on peroxide as acceptor), 44 (oxidoreductase activity, acting on single donors with incorporation of molecular oxygen), 45 (oxidoreductase activity, acting on the ch-ch group of donors), categories 39 (organismal movement) and 4 (amino acid and derivative metabolism). Categories 52 (protein kinase activity) and 27 (heparin binding) are important for characterizing both the transition from hMSC to hOST and the differences between the two intermediate states. Repeating this analysis with the second and third components identifies two additional GOs: 12 (cation transporter activity) and 42 (oxidoreductase activity, acting on paired donors, with incorporation or reduction of molecular oxygen). The proteins in this set of 12 GOs (given in Additional File 2, Directed Component Analysis Spreadsheet) provide a set of potential biomarkers of interest. Analysis of this set of proteins is provided in the discussion section.

### Three-way analysis

In this section, our goal is to identify a set of significant GeneIDs and to capture the structure within that set, taking into consideration in which categories and samples they are present. While insightful for visualization and general trends, the two-way analysis of the (Samples, GO) data involves a loss of information that limits the information revealed regarding relevant GeneIds. The DCA analysis will miss critical proteins that fall outside of the 13 categories identified. Multiway analysis fully preserves the three-way nature of the data (GeneIDs, Categories, and Samples).

**Figure 4:**
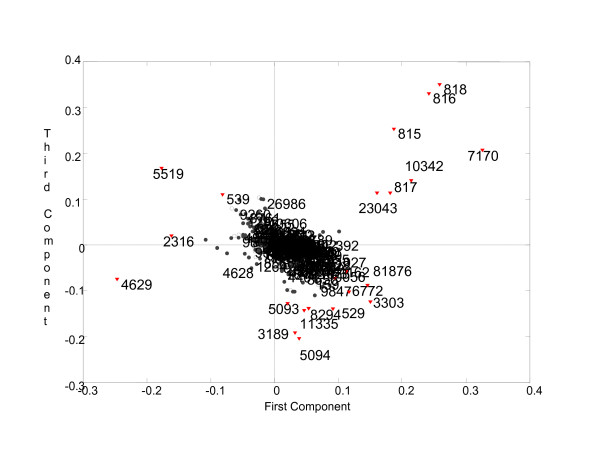
**Scatter plot of GeneIDs**. 361 GeneIDs are projected onto the space spanned by the first and third components in GeneID mode. Red color and diamond shape are used to indicate the GeneIDs considered significant and examined closely. The rest of the GeneIDs have comparatively low loading coefficients and areconsidered insignificant.

The Tucker3 [24] analysis was used to determine components for each of the three modes: Sample, GeneID, and GO. The tensor was preprocessed in the same way as in the two-way analysis: by truncating the number of proteins in each entry to at most two and by transforming the data to make hMSC the origin. We make use of the results provided by two-way analysis to determine the number of components to be extracted from each mode in the Tucker3 model. Component numbers are chosen such that the relationship observed in the sample mode coincides with the results of two-way analysis of the Samples x Categories matrix. We select components in the Category mode that best model the categories considered significant in capturing the structure in Samples mode (those having high-loading coefficients). Then core elements are inspected in order to identify the components in GeneID mode whose interaction with selected components in other modes is represented with high core values. Finally, we examine the scatter plot of the selected components (the first and third components have highest core value) in GeneID mode to understand the structure among GeneIDs. Figure 4 illustrates GeneIDs projected onto the space spanned by the first and third components of the GeneID mode. We are particularly interested in the outliers detected through loading coefficients. A set of 23 outlier GeneIDs, detected through both thresholding and statistical confidence testing, is marked in Figure 4. A table containing these proteins can be found in Additional File 3, Tensor Analysis Spreadsheet.

When we study the selected GeneIDs closely, we observe that two underlying principles govern how they spread around the plot: Samples and Categories. For instance, some outlier GeneIDs, e.g. {815, 816, 817, 818, 7170, 10342, 23043}, cluster together in the first quadrant. That is because they not only exist in the same samples (mostly Sample 1 – hMSC) but also share significant categories, i.e. Protein Kinase Activity, Transferase Activity, Transferring Phosphorus-containing Groups, etc. On the other hand, some GeneIDs are farther apart from each other if they differ in either categories or samples mode. For example, GeneIDs 529 and 539 are available in exactly the same categories but they differ in the samples in which they are present. Therefore, one of them is in the second quadrant while the other is in the fourth in Figure 4.

## Discussion

Undifferentiated hMSC, ECM-hMSC, OS-hMSC, and fully differentiated hOST express many proteins in common, yet each population also expresses distinct sets of proteins. We believe these differences allow us to discriminate between different degrees of osteogenic differentiation in hMSC, and suggest possible mechanisms for how osteogenic differentiation occurs in these cells. To provide additional information to offset the low sample size, we performed the analysis in the GO space. Our DCA established a "differentiation axis" that allows us to rank and compare intermediate states of osteogenic differentiation. This axis represents the vector accounting for the greatest variance between hMSC and hOST and gives us a new tool to uncover protein/gene expression differences that may be related to stem cell and osteoblast function. Tensor analysis in the (sample, GO, GeneID) space was used to further elucidate proteins critical for differentiation. Our approach is entirely different from, and complimentary to, more traditional comparative methods such a DNA microarray, SAGE, and EST sequence analysis [25-28]. The addition of gene ontology to the data tensor provides a critical difference between our approach and the now standard Principal Component Analysis (PCA) and more recent tensor analysis [29] for gene expression data.

Our differentiation axis begins with undifferentiated stem cells. The DCA and tensor analyses identified at least 20 proteins expressed in undifferentiated hMSC, but not in hOST, that may help define the activities of undifferentiated or partially differentiated stem cells. Some of these, such as the eukaryotic translation factors (EIF2, EIF4, EIF5), histone H4i, the lysosomal proteon pump ATP6V1E1, the peroxisomal biogenesis factor 6, asparagine synthetase, ER/Golgi transport (Rab1B), and chromobox homolog 3 participate in basic cellular activities and reflect the relatively generalized state of naïve stem cells. Perhaps of greater interest are those proteins known to play a role in directing activity of non-osteogenic cell types. For example, we identified several proteins that have been linked with immune cell activation (e.g., carboxypeptidase N, SET translocation, STAT1, CamK2G). And most interestingly, we see a number of signal transduction proteins that help define undifferentiated stem cells in our data set. For example, only unstimulated hMSC express TRAF2 and NCK interacting kinase, which regulates actin cytoskeletal organization [30], and TRK-fused gene, which mediates signaling through NF-κB in numerous cell types [31]. The most striking trend we see is the presence of four calmodulin-dependent protein kinase 2 isoforms in hMSC but none in hOST, consistent with our previous suggestion that calmodulin-based signaling is a potential hallmark of undifferentiated hMSC [32]. Collectively, this expression profile supports the notion that hMSC are more like "generic" cells than their differentiated counterparts, though a functional definition of what constitutes a stem cell has yet to be established [33].

At the opposite end of our axis are fully differentiated osteoblasts. We identified 13 proteins that are expressed in osteoblasts but not in stem cells and which our analyses suggest contribute to the osteoblast phenotype. For some of these proteins, the connection to osteoblast function is fairly clear: fibronectin is expressed in the matrix of developing and mature bone and promotes the early stages of bone formation (e.g., [34]), and vitronectin promotes osteogenic differentiation of hMSC [13]. Several isoforms of dihydrodiol dehydrogenase, which metabolizes progesterone, are also found in hOST, reflecting the importance of steroid hormones in skeletal growth and maintenance [35]. Others are less obvious – e.g., superoxide dismutase 1 and glutathione peroxidase 1, which provide protection against oxidative stress, may serve an important maintenance role during osteoblast differentiation. Also, peroxiredoxin 5 protects cartilage cells from oxidative stress and maintains collagen synthesis [36] – perhaps these proteins perform a similar function during collagen deposition by osteoblasts.

Along the middle of our axis lie the treated hMSC, and we think that these represent intermediate states of osteogenic differentiation. Our analyses identified five proteins that may distinguish these states from naïve stem cells or osteoblasts and may uncover clues as to how osteogenic differentiation takes place in hMSC. Interestingly, the heavy chain of smooth muscle myosin is in this group: recently, Discher's group demonstrated that non-muscle myosin II mediates cell lineage specification in these cells [10], suggesting that other myosins may play a role in determining cell lineage specificity in response to ECM binding. Consistent with this hypothesis, filamin A is also found in this group. Filamin A crosslinks cortical actin filaments, serves a mechanoprotective function in response to tensile strain [37], and is controlled by calcium/calmodulin signaling [38]. Because calcium/calmodulin signaling appears to decline during osteogenesis in hMSC, filamin A activity may represent an early step towards lineage commitment in these cells. Two other proteins that appear in all of our cell populations (except hOST) may also serve as markers of an intermediate state: glucose phosphate isomerase (also known as Autocrine Motility Factor) regulates cell growth and migration of numerous cell types [39], and activin A receptor (type IIB) supports growth of germ cells in the developing human embryo [40]. As stem cells are a continually self-renewing population, whereas osteoblasts are much less proliferative, control of growth may be a crucial step in moving away from the stem cell phenotype.

What is especially striking is that two different protein expression patterns occur in these intermediates: that induced by contact with ECM proteins and that induced by OS media. Our DCA identified three proteins that distinguish the ECM-directed route from the OS-directed route, and all of these (two subunits of proline-4 hydroxylase and lysine hydroxylase 2) are involved in collagen synthesis and processing. These proteins are found in hOST and all ECM-treated populations, but not in OS, suggesting that collagen synthesis in hMSC is preferentially driven by ECM contact. Consistent with this, we and others have found that plating hMSC on collagen I stimulates additional collagen synthesis [13, e.g., 41, 20]. Conversely, OS-treated hMSC express four proteins involved in steroid metabolism (dihydrodiol dehydrogenase 1 and 2; 3-alpha hydroxysteroid dehydrogenase, type II; peroxisomal trans-2-enoyl-CoA reductase) that are not found in ECM-treated populations. Only two of these proteins appear in hOST. Perhaps this is not surprising, since OS media contains the steroid analog dexamethazone, but it illustrates an important point: hMSC can be stimulated to undergo osteogenic differentiation by two seemingly independent routes – one driven by ECM signaling and one by steroid hormone signaling.

## Conclusion

Our three-mode tensor-based proteomic analysis, based on gene ontologies and validated using hMSC plated on laminin-5, has revealed two independent mechanisms by which human mesenchymal stem cells (hMSC) undergo osteogenic differentiation. The differences between the proteomic profiles of OS-stimulated hMSC and ECM-hMSC characterize different transitional phenotypes en route to becoming osteoblasts. One of these is driven by ECM signaling and the other by steroid hormone signaling. In addition, stimulation with ECM proteins results in a more osteoblast-like phenotype than that resulting from stimulation with OS media. These results, arrived at through interdisciplinary means, contribute to a better understanding of osteogenesis and thus, we hope, eventually to improved treatment for relevant diseases and tissue damage.

## Methods

### Materials

Bovine collagen I and vitronectin were purchased from Chemicon (Temecula, CA). All other reagents and cell culture supplies were obtained as previously described [15,16]. Protein profiles from hMSC, hMSC cultured in OS medium, and hOST were accessed from our online database described in [11].

### Culture of hMSC

Cryopreserved hMSC were routinely passaged as previously described [11]. To collect protein expression profiles from hMSC stimulated with ECM proteins, cells were grown in hMSC growth media in tissue culture dishes coated with 20 μg/ml collagen I or 20 μg/ml vitronectin, or grown on tissue culture dishes containing laminin-5 deposited by 804G rat bladder carcinoma cells as previously described [16].

### 2D LC-MS/MS

Preparation of whole cell lysates (collected after 16 days in culture) from hMSC cultured on ECM proteins for 2D LC-MS/MS was performed as previously described [11]. Briefly, protein pellets were dissolved in 100 mM Tris-HCl, pH 8.5, 5 mM tributyl phosphine, and 6.4 M urea. The protein mixtures were incubated for 30 min at 37°C followed by the alkylation in 15 mM iodoacetamide. Reactants were then diluted six-fold and subjected to tryptic digestion overnight at 37°C. The reaction was stopped with the addition of 90% formic acid, and the resultant peptides were then concentrated with C18 cartridges and exchanged into 5% acetonitrile, 0.4% formic acid, and 0.005% heptafluorobutylic acid (HFBA). Samples (120 μg protein) were analyzed in duplicate using an analytical system consisting of a CapLC autosampler, CapLC pumps, stream selector, Z-spray probe, and a quadruple time-of-flight mass (TOF) spectrometer. The setup was configured with a polysulfoethyl strong cation exchange (SCX) column (320 μm ID X 80 mm, packed with 20 μm POROS 20 HS from Applied Biosystems) in series with a desalting column (300 μm ID X 5 mm, packed with a C18 stationary phase from Thermo Quest Inc.) and a reverse-phase C18 column (75 μm ID X 110 mm, packed with BetaBasic C18 resin from ThermoHypersil Keystone) for two-dimensional separations. For elution, solvent A9 consisted of 3% CH3CN, 0.4% acetic acid, and 0.005% HFBA; solvent B was 90% CH3CN, 0.4% acetic acid, and 0.005% HFBA. Tryptic digests were loaded onto the 2D LC-MS/MS system under pressure. Peptides in 10 mM NaCl solution were first absorbed onto the SCX column; the peptides in the flow through were washed onto the reverse-phase peptide-trapping column where they were concentrated and desalted. First-stage separation was achieved by eluting the SCX column with 20 μl each of 0, 10, 20, 30, 40, 50, 60, 70, 80, 90, 100, 120, 140, 150, 160, 200, 250, 300, 500, and 830 mM KCl. For second-stage separation, each of the eluates was separated on a reverse-phase column by the application of a series of mobile phase-B gradients (1–10% B in 5 min, 10–15% B in 25 min, 15–20% B in 15 min, 20–45% B in 10 min, 45–80% B in 5 min). The separated peptides were characterized by their mass and sequence data (MS/MS). To load samples, a picofrit column was connected directly to a Q-TOF 2 Zsprayer. Approximately 2,100 volts were applied to the spray tip. Approximately 5 psi of nebulizing gas was introduced around the spray tip to aid the electrospray process. A splitter gave a resultant flow through the analytical column of 200 nl/min with the pump programmed to deliver a flow of 6.5 μl/min. The mass spectrometer was operated in a data-dependent acquisition mode whereby, following the interrogation of MS data, ions were selected for MS/MS analysis based on their intensity and charge state. The detection threshold for this instrument is 200 pmol per protein. Collision energies were chosen automatically based on the m/a and charge-state of the selected precursor ions. Ion data were compared to the National Center for Biotechnology Information (NCBI) nonredundant homo sapiens database using the Proteinlynx 1.1 Global Server program.

### Data Preparation

945 different proteins were identified by 2D LC MS/MS; 765 different proteins were identified with accession numbers (in Ensemble, Refseq, or Trembl format) and a protein name. Each protein was also matched to its GeneID at NCBI [42], if available, by accessing the European Bioinformatics Institute [43], downloading the ipi.HUMAN.xrefs.gz IPI dataset [44], and matching each accession number to its corresponding gene ID number. Proteins that were not assigned a GeneID using this method were searched by hand using the online search function (IPI Quick Search) and the search feature located at Entrez Gene by entering available accession numbers and the protein name.

A given protein as identified by the GeneID may have multiple accession numbers. The number of accession numbers per protein was truncated to 2. Since laminin-5 hMSC proteomic analysis was used as validation, only GeneIDs found in the first five samples were considered. There are 555 such GeneIDs, corresponding to 758 proteins.

To determine protein functional relationships within and between each data set, we used the Database for Annotation, Visualization and Integrated Discovery (DAVID) [45] to annotate proteins identified by GeneID with their gene ontologies. The GeneIDs of proteins in the six samples were categorized in Biological Process and Molecular Function gene ontology categories using the GO Chart feature offered by DAVID 1.0 with settings of intermediate coverage and specificity (Level 3) with a minimum of 4 GeneIDs as done in [15,16]. The GO categories were determined for each data set and then unioned to form a complete list. DAVID was set at intermediate coverage and specificity (Level 3) with a minimum of 4 GeneIDs. The 69 GO categories used in this study are given in Additional File 1.

A relational database management system (DBMS) was used to develop both the gene ontology analysis and the further protein classifications. Reports and queries were written and generated, using the DBMS facilities and additional programming, to help produce the statistics reported in this paper and to help produce the 3-dimensional Proteomics Array described next.

Each entry in the three-way Proteomics Array, X_ijk_, represents the number of accession identifiers observed for the i^th ^protein available in the j^th ^category for the k^th ^sample. We apply several preprocessing steps compatible with those of two-way analysis. First, proteins that are available in all samples and the ones that do not exist in any of the samples are removed. The dimension of X is 361 × 69 × 5 after the elimination of these proteins. Second, each entry is truncated to 0, 1, or 2. For the two-way model, we first add the data across the GeneID mode to form a Sample x Category matrix, then divide each GO vector by its sum, and finally center the data by subtracting the hMSC vector. For the multi-way model, we center the data with respect to hMSC, which means that the slice or matrix corresponding to hMSC is subtracted from the slices corresponding to other samples. Note that as validation, the two-way and multi-way analyses are performed using only the first five samples and then the results for laminim-5 hMSC are projected onto the components defined by the first five cell populations.

### Two-way analysis: Directed Component Model

Consider matrix X consisting of 69 categories as rows and 5 features (experiments) as columns. Call each column x_j _so that X= [x_1 _x_2 _x_3 _x_4 _x_5_]. Each entry X_ij _represents the active proteins in category i found in experiment j. We seek a linear model for the x_j _as follows:

*x*_*j *_= *d*_*offset *_+ *c*_*directed *_*d*_*directed *_+ *c*_sec*ond *_*d*_sec*ond *_+ *c*_*third *_*d*_*third *_+ *c*_*fourth *_*d*_*fourth*_.

The vectors *d *are directions that make up the columns of X and the scalars *c *are distances in these directions. Each direction is now discussed in turn.

To begin, the first direction is the starting point for osteogenesis, that is, stem cells. Hence *d*_*offset *_= *x*_1_, which is hMSC. Next, we are interested in the direction that connects stem cells to osteoblasts. Hence *d*_*directed *_= *x*_5 _- *x*_1_, which hOST less hMSC. For each experiment, *c*_*directed *_is the extent to which that experiment is similar to osteoblasts along this route. These constants were plotted in Figure 1, providing an implicit ranking of experiments. We see that the three ECM-stimulated populations are closer to osteoblasts than are the OS media-stimulated populations.

The remaining directions {*d*_sec*ond*_, *d*_*third*_, *d*_*fourth*_} are chosen such as to maximize the remaining variability subject to being uncorrelated (perpendicular) with the previous directions. That is, these directions are the principal components of the remaining space. The constants thereof {*c*_sec*ond*_, *c*_*third*_, *c*_*fourth*_} indicate how far away from the direct path linking stem cell to osteoblast an experiment is. The directed component accounts for 26% of the variability while the second, third, and fourth components account for 41%, 22%, and 10% of the variability respectively in the first five experiments.

### Multi-way analysis: Tucker3 Model

We model the three-way Proteomics Array, X ∈ ***R ***^IxJxK^, as in Equation 1, using a Tucker3 [24] model, which is one of the most common analysis techniques in the multi-way literature.

Xijk=∑r=1R∑q=1Q∑p=1PGijkAipBjqCkr+Eijk
 MathType@MTEF@5@5@+=feaafiart1ev1aaatCvAUfKttLearuWrP9MDH5MBPbIqV92AaeXatLxBI9gBaebbnrfifHhDYfgasaacH8akY=wiFfYdH8Gipec8Eeeu0xXdbba9frFj0=OqFfea0dXdd9vqai=hGuQ8kuc9pgc9s8qqaq=dirpe0xb9q8qiLsFr0=vr0=vr0dc8meaabaqaciaacaGaaeqabaqabeGadaaakeaacqWGybawdaWgaaWcbaGaemyAaKMaemOAaOMaem4AaSgabeaakiabg2da9maaqahabaWaaabCaeaadaaeWbqaaiabdEeahnaaBaaaleaacqWGPbqAcqWGQbGAcqWGRbWAaeqaaOGaeeyqae0aaSbaaSqaaiabbMgaPjabbchaWbqabaGccqqGcbGqdaWgaaWcbaGaeeOAaOMaeeyCaehabeaakiabboeadnaaBaaaleaacqqGRbWAcqqGYbGCaeqaaOGaey4kaSIaemyrau0aaSbaaSqaaiabdMgaPjabdQgaQjabdUgaRbqabaaabaGaeeiCaaNaeeypa0JaeeymaedabaGaeeiuaafaniabggHiLdaaleaacqqGXbqCcqqG9aqpcqqGXaqmaeaacqqGrbqua0GaeyyeIuoaaSqaaiabbkhaYjabb2da9iabbgdaXaqaaiabbkfasbqdcqGHris5aaaa@5F12@

**Figure 5:**
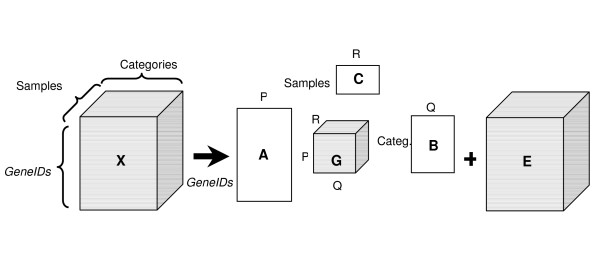
**Three-way Proteomics Array and Tucker3 Model**. Proteomics Array with GeneID, Category, and Sample modes is modeled with [P Q R] – Tucker3 model. A, B, and C are component matrices corresponding to GeneID, Category, and Sample modes, respectively. G is the core array and E contains the residuals for each entry in X.

Here P, Q, and R indicate the number of components extracted from the  geneID, GO and sample modes, respectively. A ∈ ***R ***^IxP^, B ∈ ***R ***^JxQ^, and C ∈ ***R ***^KxR ^are orthogonal component matrices. G ∈ ***R ***^PxQxR ^is the core array and E ∈ ***R ***^IxJxK ^represents the error term. A Tucker3 model with orthogonality constraints on component matrices is also called Higher-Order Singular Value Decomposition (HOSVD) [46]. Figure 5 illustrates the three-way Proteomics Array and how it is modeled using a Tucker3 model. In our analysis, we make use of the PLS Toolbox [47] and for detailed information on the Tucker3 model and other multiway analysis techniques, the reader is referred to [48].

## List of Abbreviations

Human mesenchymal stem cells (hMSC)

osteogenic stimulant (OS)

human osteoblasts (hOST)

Entrez gene ID (GeneID)

GO (Gene Ontology)

ECM stimulated hMSC (ECM-hMSC)

Directed Component Analysis *(DCA)*

## Authors' contributions

KB, BY, and GP conceived the study and drafted portions of the manuscript. CB prepared and analyzed data. EA and BY carried out the tensor analysis. RK and GP created the cultures and carried out the 2D LC-MS/MS. KB and SV acquired, prepared, and analyzed data. SV also drafted part of the manuscript. All authors read and approved the final manuscript.

## Supplementary Material

Additional file 1 The 69 GO (Gene Ontology) categories used in this study, listed alphabetically Click here for file

Additional file 2 Directed Component Analysis Data. Gene ontologies, GeneIDs, symbols, names, and distinct protein counts for the six samples (hMSC, OS-hMSC, col-hMSC, FN-hMSC, hOST, and LN5-hMSC). Click here for file

Additional file 3 Tensor Analysis Data. Contains outliers detected by choosing the GeneIDs outside the 95% confidence ellipse. Each GeneID is accompanied by its symbol, name, and the counts of distinct proteins found in the six samples (hMSC, OS-hMSC, col-hMSC, FN-hMSC, hOST, and LN5-hMSC). Click here for file
