# Supplementary material for: Proteomics reveals multiple routes to the osteogenic phenotype in mesenchymal stem cells
Source: BMC Genomics. 2007 Oct 19;8:380. doi: 10.1186/1471-2164-8-380 (PMC2148065; doi:10.1186/1471-2164-8-380)
Supplement: Additional file 1 — The 69 GO (Gene Ontology) categories used in this study, listed alphabetically [file 1471-2164-8-380-S1.doc]

**Supporting Information I:**

Table 1: The 69 GO (Gene Ontology) categories used in this study, listed alphabetically.

| **Categories** |
| --- |
| 1: alcohol metabolism  2: alpha-type channel activity  3: amine metabolism  4: amino acid and derivative metabolism  5: biosynthesis  6: blood coagulation  7: calcium ion binding  8: calmodulin binding  9: carbohydrate metabolism  10: carbon-oxygen lyase activity  11: catabolism  12: cation transporter activity  13: cell adhesion  14: cell death  15: cell growth and/or maintenance  16: cell motility  17: cell-cell signaling  18: cellular morphogenesis  19: coenzyme and prosthetic group metabolism  20: cytokine activity  21: cytoskeletal protein binding  22: disulfide oxidoreductase activity  23: dna binding  24: electron transport  25: energy pathways  26: gtpase activator activity  27: heparin binding  28: hydrolase activity, acting on acid anhydrides  29: hydrolase activity, acting on carbon-nitrogen (but not peptide) bonds  30: hydrolase activity, acting on ester bonds  31: immune response  32: intramolecular isomerase activity  33: ligase activity, forming carbon-nitrogen bonds  34: lipid metabolism  35: magnesium ion binding  36: neurophysiological process  37: nucleobase, nucleoside, nucleotide and nucleic acid metabolism  38: organic acid metabolism  39: organismal movement  40: organogenesis  41: oxidoreductase activity, acting on ch-oh group of donors  42: oxidoreductase activity, acting on paired donors, with incorporation or reduction of molecular oxygen  43: oxidoreductase activity, acting on peroxide as acceptor  44: oxidoreductase activity, acting on single donors with incorporation of molecular oxygen  45: oxidoreductase activity, acting on the ch-ch group of donors  46: oxygen and reactive oxygen species metabolism  47: peptidase activity  48: phosphorus metabolism  49: primary active transporter activity  50: protease inhibitor activity  51: protein domain specific binding  52: protein kinase activity  53: protein metabolism  54: purine nucleotide binding  55: regulation of body fluids  56: regulation of cell growth  57: regulation of cell proliferation  58: response to external stimulus  59: response to stress  60: rna binding  61: signal transduction  62: small gtpase regulatory/interacting protein activity  63: sugar binding  64: transcription factor binding  65: transferase activity, transferring acyl groups  66: transferase activity, transferring phosphorus-containing groups  67: transition metal ion binding  68: translation factor activity, nucleic acid binding  69: translation initiation factor activity |
